# 724. LDBIO *Toxoplasma* Test in the United States and Beyond: Review of the Device’s Performance on U.S. Samples and its Ability to Avoid False Positives

**DOI:** 10.1093/ofid/ofab466.921

**Published:** 2021-12-04

**Authors:** Andrew Grose, Rima McLeod

**Affiliations:** 1 University of Chicago Pritzker School of Medicine, Chicago, Illinois; 2 University of Chicago Medical Center, Chicago, Illinois

## Abstract

**Background:**

Part of an essential “toolbox” to eliminate *Toxoplasma gondii* infection is prompt recognition of acute infection acquired during gestation, in order to initiate treatment for congenital toxoplasmosis (CT). From conception to one month post-partum, screening seronegative pregnant women monthly for antibody to the parasite enables treatment that prevents trans-placental transmission of newly acquired maternal *Toxoplasma*, or that attenuates signs and symptoms of CT. Tests that are highly sensitive and specific—and that meet the other World Health Organization ASSURED criteria for diagnostics—are very useful for this kind of screening. Herein, we evaluated the accuracy of a test that meets these criteria—the LDBIO *Toxoplasma* ICT IgG-IgM device (LDBIO)—and whether it eliminated difficulties of other tests with false positive IgM results.

World Health Organization A.S.S.U.R.E.D. criteria

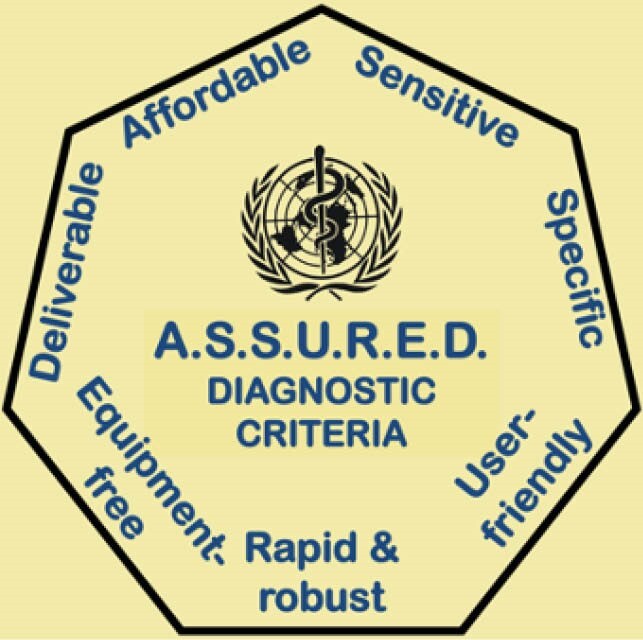

These are criteria for ideal screening or diagnostic tests, as described in a September 2017 paper in the Bulletin of the World Health Organization. Our study focused mostly on sensitivity and specificity for the LDBIO immunochromatography test for IgG and IgM specific to Toxoplasma gondii.

**Methods:**

Both parts of this study examined results generated by the LDBIO device—a point-of-care immunochromatography test for *Toxoplasma* IgG and IgM—using serum and whole blood samples. With whole blood, thirty microliters were collected using a glass micro hematocrit tube. With both sera and whole blood, samples were loaded into the well of the LDBIO device, which took 20 minutes to generate results. In the first part of this study, we summarized results from three published U.S. studies and added new data from an ongoing clinical trial at the University of Chicago Medical Center (UCMC). In the second part of this study, we compiled data on how the LDBIO device performed on a total of 69 samples from U.S. and French studies that had led to false positive results when tested with commercially available comparator tests. Four of these false positives came from the UCMC trial.

UCMC Feasiblity Study Flowchart

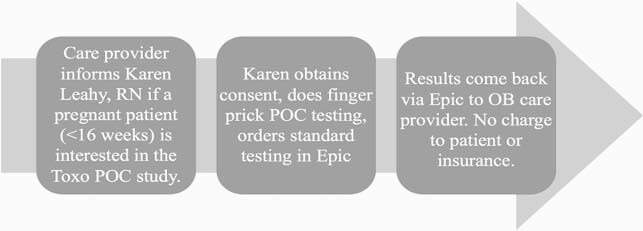

Flowchart for ongoing feasibility study on the LDBIO device at the University of Chicago Medical Center. Data from this study may inform whether the LDBIO test—which already has the CE Mark for use in Europe—will receive 510(k) approval from the Food and Drug Administration in the U.S.

Steps for Using LDBIO Device

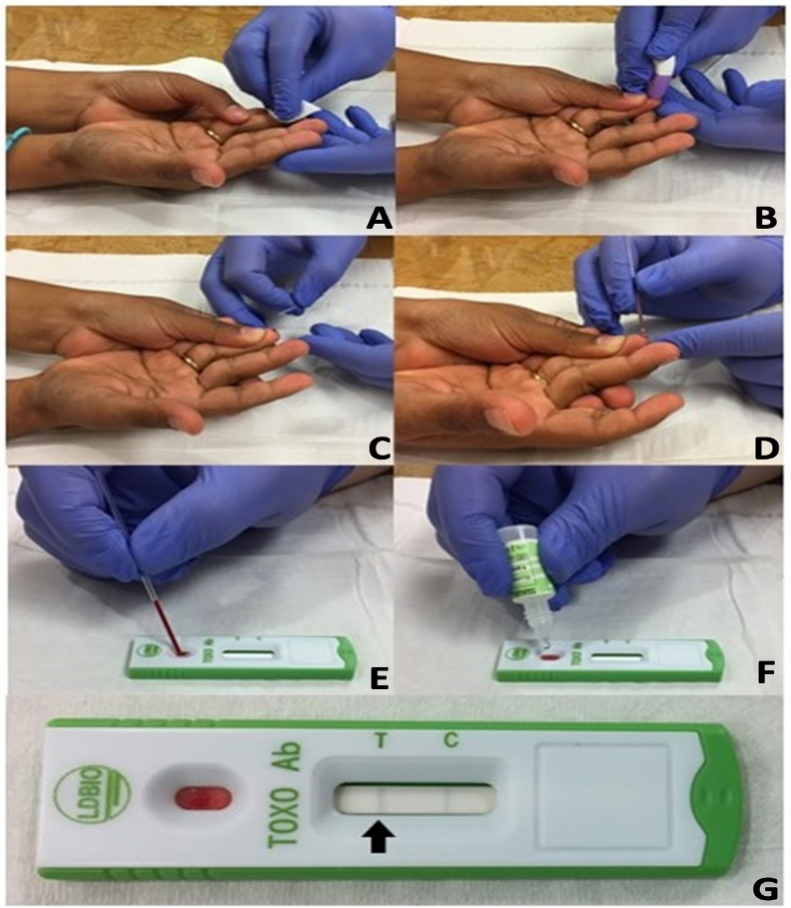

(A,B) Clean fingertip; prick with lancet (if collecting whole blood only) (C,D) Collect 30 uL in capillary tube (WB only) (E,F) Apply serum or blood sample to well; add four drops buffer and wait about 20 minutes (G) How to interpret results: black line under “T” corresponds to IgG and/or IgM to T. gondii

**Results:**

LDBIO had only one false negative for a total of 664 samples from three earlier U.S. studies and the UCMC feasibility study. Meanwhile, out of 69 total false positives from various non-reference laboratory comparator tests, such as the Bio-Rad Platelia and Siemens kits, the LDBIO generated zero false positives.

LDBIO's Performance on U.S. Samples Since 2014

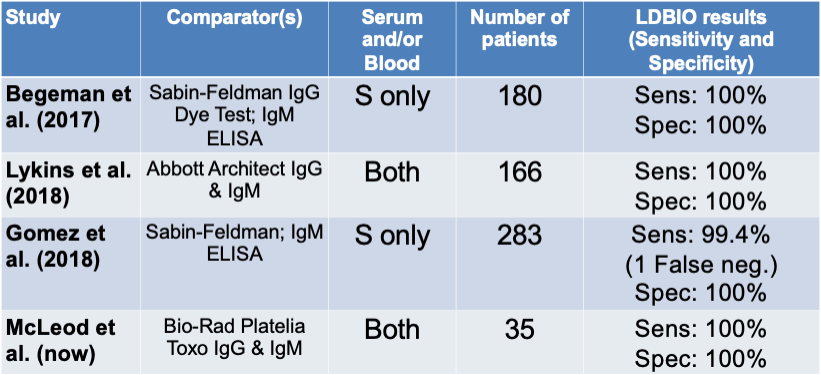

In all four U.S. studies (total 664 patients), the LDBIO device generated one false negative result and zero false positive results.

LDBIO vs. Comparator Tests Since 2017

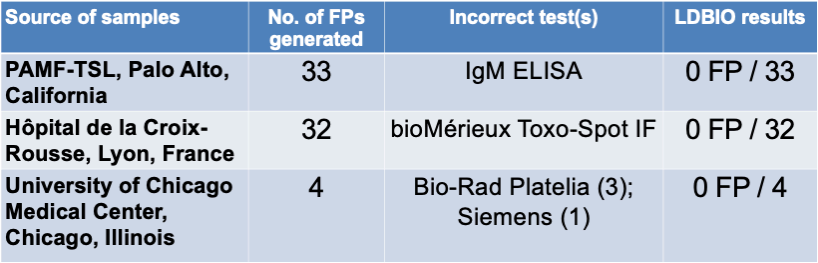

In these three clinical settings (69 total samples), LDBIO correctly avoided generating the same false positive that had been generated by a test already cleared for widespread use in the U.S. or France.

**Conclusion:**

As LDBIO shows high sensitivity and specificity and can avoid confounding false positive results, this device merits consideration as a high-quality screening test that can assist public health efforts to improve CT care worldwide.

Countries Working to Implement Regular Prenatal Screening for CT Prevention

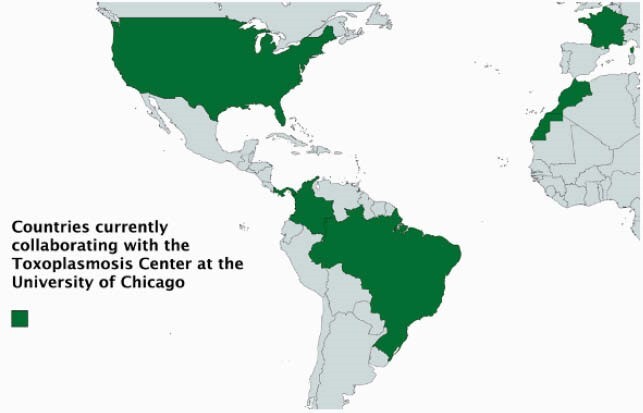

The countries in green represent countries currently working with the University of Chicago to implement regular prenatal screening programs for Toxoplasma gondii: U.S., Panama, Colombia, Brazil, Morocco, and France. Screening programs in all six countries rely on low-cost, highly-accurate screening technology that meets the WHO's ASSURED criteria. The LDBIO test -- which is already in use in France -- may become a usable resource in the other five countries if it gains FDA approval.

**Disclosures:**

**All Authors**: No reported disclosures

